# Combined value of left ventricular ejection fraction and the Model for End-Stage Liver Disease (MELD) score for predicting mortality in patients with acute coronary syndrome who were undergoing percutaneous coronary intervention

**DOI:** 10.1186/s12872-018-0782-8

**Published:** 2018-03-02

**Authors:** Tuncay Kırıs, Eyüp Avcı, Aykan Çelik

**Affiliations:** 1 Department of Cardiology, Izmir Katip Celebi University, Ataturk Training and Research Hospital, 35360 Izmir, Turkey; 2 Department of Cardiology, Balikesir University Faculty of Medicine, 10345 Balikesir, Turkey

**Keywords:** MELD score, LVEF, Acute coronary syndromes, Mortality

## Abstract

**Background:**

The purpose of the study was to investigate whether the addition of left ventricular ejection fraction (LVEF) to the MELD score enhances the prediction of mortality in patients with acute coronary syndrome (ACS) undergoing percutaneous coronary intervention (PCI).

**Methods:**

This retrospective study analyzed 846 consecutive patients with ACS undergoing PCI who were not receiving previous anticoagulant therapy. The patients were grouped as survivors or non-survivors. The MELD score and LVEF were calculated in all patients. The primary end point was all-cause death during the median follow-up of 28 months.

**Results:**

During the follow-up**,** there were 183 deaths (21.6%). MELD score was significantly higher in non-survivors than survivors (10.1 ± 4.4 vs 7.8 ± 2.4, *p* <  0.001). LVEF was lower in non-survivors compared with survivors (41.3 ± 11.8% vs. 47.5 ± 10.0%, *p* <  0.001). In multivariate analysis, both MELD score and LVEF were independent predictors of total mortality. (HR: 1.116, 95%CI: 1.069–1.164, *p* <  0.001; HR: 0.972, 95%CI: 0.958–0.986, *p* <  0.001, respectively). The addition of LVEF to MELD score was associated with significant improvement in predicting mortality compared with the MELD score alone (AUC:0.733 vs 0.690, *p* <  0.05). Also, the combining LVEF with MELD score improved the reclassification (NRI:24.6%, *p* <  0.001) and integrated discrimination (IDI:0.045, *p* <  0.001) of patients compared with MELD score alone.

**Conclusions:**

Our study demonstrated that the combining LVEF with MELD score may be useful to predict long-term survival in patients with ACS who were undergoing PCI.

**Electronic supplementary material:**

The online version of this article (10.1186/s12872-018-0782-8) contains supplementary material, which is available to authorized users.

## Background

Acute coronary syndromes (ACSs) which encompass unstable angina (UA) together with non-ST-elevation myocardial infarction (NSTEMI) and ST-elevation myocardial infarction (STEMI) are the leading cause of death and high morbidity worldwide [1, 2]. Various biomarkers and risk stratification scores have been developed and used to predict prognosis of these patients [3, 4]. The Model for End-stage Liver Disease (MELD) score including serum creatinine (sCr), total bilirubin (TB), and international normalized ratio (INR) are commonly used to estimate prognosis among patients with chronic liver diseases of different etiologies [5]. In addition, this score can be effective in the prediction of nonoperative outcomes, such as evaluating risk for patients with congestive heart failure [6].

Serum creatinin and total bilirubin levels measured at hospital admission seem to be associated with mortality in patients with ACS [7, 8]. Similarly, it has recently been shown that an increase INR in the absence of anticoagulant therapy is associated with mortality in patients with both acute pulmonary embolism (PE) and heart failure [9, 10]. Left ventricular systolic dysfunction has been associated with increased mortality after ACSs [11].

As both MELD score include the above-mentioned laboratory parameters and left ventricular ejection fraction (LVEF) related to mortality in cardiovascular diseases, we aimed to investigate whether the addition of LVEF to MELD score creates additional prognostic value for all-cause mortality in patients with ACS treated with percutaneous coronary intervention (PCI) who were not on anticoagulant therapy.

## Methods

### Study population

We retrospectively evaluated 910 consecutive patients with ACS treated with PCI from april 2008 and July 2015. To be enrolled in the study, patients had to have angiographically proven ACS and baseline INR, sCr, and TB measurements. Nine patients with incomplete data, two with a history of liver cirrhosis, 14 who had received anticoagulant therapy (vitamin-K antagonists, direct thrombin inhibitors, direct factor Xa inhibitors, or enoxaparin), 29 patients with right ventricular dilatation/failure and moderate to severe tricuspid regurgitation were excluded from the analysis**.** Consequently, the final study population consisted of 846 patients. They were divided into survivors (*n* = 663) and non-survivors (*n* = 183) based on the total mortality at follow-up. The local ethics committee approved the study. The study conforms to the Declaration of Helsinki.

### Blood sampling and calculation of MELD score

All measurements of INR, sCr, and TB were performed at the presentation of the patients prior to the initiation of anticoagulant therapy and coronary angiography. The blood-collection tubes contained 3.2% sodium citrate (0.5 ml citrate, 4.5 ml blood) for INR, measurement. Samples were immediately centrifuged for routine testing, and analysis was performed within 1 h after sampling. INR was measured using the reagent HemosIL RecombiPlasTin 2G (Instrumentation Laboratory, Bedford, MA, USA). Complete blood count was determined via an Abbott Cell-Dyn 3700 autoanalyzer using commercial assay kits (Abbott Diagnostic, CA, US). Biochemical measurements were performed using Siemens Healthcare Diagnostic Products kits and calibrators (Marburg, Germany).

The standard MELD score was calculated by using the following formula: 11.2 x (ln INR) + 0.378 x (ln total bilirubin) + 0.957 x (ln creatinine) + 0.643 [6].

### Echocardiographic analysis

Echocardiographic examinations were performed for all patients. The left ventricular ejection fraction (LVEF) was calculated after measuring the end-diastolic and end-systolic left ventricul (LV) volumes in the apical four-chamber and two-chamber views using the modified Simpson’s method.

Tricuspid regurjitation (TR) severity was quantified and classified on an ordinal scale as absent, mild, moderate, and severe. To estimate of right atrial (RA) pressure during echocardiography, we used 2-dimensional and Doppler imaging characteristics of the inferior vena cava and hepatic veins and graded as 5, 10, 15, and 20 mmHg. Righ ventricle (RV) systolic pressure was calculated as 4 times the square of the peak trans–tricuspid valve systolic regurgitant velocity (according to the simplified Bernoulli equation) plus the estimated RA pressure [12].

RA and RV enlargement and RV systolic function were semiquantitatively described as normal, mild, moderate, or severe enlargement or dysfunction in accordance with an ordinal qualitative scale based on visual assessment [12].

### Treatment

All coronary angiography and PCI procedures were performed via the transfemoral approach by experienced interventional cardiologists. Both the UA and NSTEMI patients underwent coronary angiography with subsequent PCI within the first 48 h. Primary PCI for STEMI was performed according to the current guidelines [13]. The diagnosis of CAD was confirmed by coronary angiography in all patients and consisted of documentation of a significant disease (defined as coronary stenoses ≥50% luminal narrowing in at least one of the major coronary arteries, or an infarct-related artery). Multivessel disease was defined as at least 50% diameter stenosis of two or more epicardial coronary arteries, or left main by visual estimation. Angiographic data of the patients were evaluated from catheter laboratory records. All patients were treated according to good clinical practice and the current guidelines [13, 14]. The type of stent and the use of thrombectomy devices, predilation, poststenting adjunctive balloon inflation, intravascular ultrasound, intra-aortic balloon counterpulsation, or glycoprotein IIb/IIIa inhibitors were all left to the operators’ discretion. Both aspirin (100 mg/day) and clopidogrel (75 mg/day) or prasugrel (10 mg/day) or tigacrelor (90 mg twice daily) were maintanied for at least 12 months, followed by indefinite single antiplatelet theraphy in our study. Beta-blockers, angiotensin-converting enzyme inhibitors, and statins were administered according to the European Society of Cardiology guidelines [13, 14].

### Definition

According to the criteria of the universal definition of myocardial infarction, diagnosis was established in the presence of an increasing/decreasing pattern in cardiac troponin I values, with at least one measurement above the 99th percentile together with evidence of myocardial ischemia [15]. Additionally, myocardial infarction was classified as STEMI or NSTEMI according to current guidelines [13, 14]. STEMI involves the presence of (1) ST-segment elevation consistent with myocardial infarction of ≥2 mm in adjacent chest leads and/or ST-segment elevation of ≥1 mm in two or more standard leads or new left bundle branch block (LBBB) and (2) positive cardiac necrosis markers. Diagnosis of NSTEMI was established in accordance with current guidelines. Including typical chest pain, serial increased levels of cardiac biomarkers and diagnostic electrocardiographic changes without ST elevation. Furthermore, UA involves (1) the absence of ST-segment elevation consistent with MI or new LBBB, (2) the presence of negative cardiac necrosis markers, and (3) the presence of angina pectoris (or an equivalent type of ischemic discomfort) with any one of the following three features: (a) prolonged (> 20 min) angina occurring at rest, (b) new-onset angina of at least Canadian Cardiovascular Society (CCS) class III severity, or (c) recent acceleration of angina reflected by an increase in severity of at least one CCS class to at least CCS class III [14]. Cardiovascular risk factors (arterial hypertension, diabetes, hypercholesterolemia, and smoking) were defined according to the accepted current criteria.

The primary study end point was defined as occurrence of all-cause total mortality during the median follow-up of 28 months. In addition, cardiac death, myocardial reinfarction, stroke/transient ischemic attack (TIA), target-vessel revascularization (TVR), and heart-failure admission were assessed. Reinfarction was defined according to the third universal definition of myocardial infarction [15]. TVR was defined as any revascularization procedure, including by-pass surgery, involving the initially treated artery. Stroke/ TIA was defined as an acute neurological deficit accompanied by brain imaging compatible with a recent ischemic or hemorrhagic event. Bleeding events were defined using the criteria of the Academic Research Consortium definition [16].

### Follow-up

The patients were followed for clinical events such as deaths, MI, stroke, and heart failure during the median follow-up of 28 months. Follow-up data were obtanied from hospital records or by interviewing (in person or by telephone) patients, their families, or their personal physicians.

### Statistical analysis

Continuous variables were expressed as mean ± standard deviation, and categorical variables were expressed as number of subjects with percentage of total number. Comparison of parametric values between the two groups was performed using Student’s t-test or the Mann-Whitney U-test, as appropriate. A chi-squared test was used to compare categorical variables between the groups. The cumulative survival curves for total mortality were estimated with Kaplan-Meier plots. A log-rank test was used to analyze the significant differences in survival curves. A multivariate Cox regression analysis was performed to identify independent predictors for the primary end point. Factors entered into the multivariate model comprised those with *p*-values < 0.1 from the univariate analysis and variables with known prognostic value. The predictive values of MELD score and a combination of LVEF and MELD score were estimated by comparing the areas under the receivers operating characteristic (ROC) curve. DeLong’s test was used to compare the AUC from each of models [17], which were analysed by use of Analyse-it software programme. Morever, the increased discriminative value after the addition of LVEF to MELD score was also estimated using the Net Reclassification Improvement (NRI) and Integrated Discrimination Improvement (IDI) [18]. Two-sided *p*-values < 0.05 were considered statistically significant. Statistical tests were performed with SPSS version 16 (SPSS Inc., Chicago, IL, USA).

## Results

### Baseline characteristics

The mean age was 62.2 ± 12.3 years. Of the 846 patients, 629 (74%) were males and 217 (26%) were females. The median follow-up period was 28 months (inter-quartile range 25th and 75th percentile: 13 to 44 months). The baseline characteristics of the study patients are presented Table 1. Subgroup analysis according to both gender and age was performed. For age, age was categorized as < 65, and ≥ 65 years. Also, this analysis was presented as Additional file 1: Tables S6 to S8 (for gender), and Additional file 2: Tables S9 to S11 (for age).

**Table 1:** Baseline characteristics of the study population

Variable	Survivors (*n* = 663)	Non-survivors (*n* = 183)	*P*-value
Age (year)	62 ± 12	67 ± 12	< 0.001
Female n (%)	158 (24)	59 (32)	0.021
History of HF n (%)	11 (2)	15 (8)	< 0.001
Hypertension n (%)	301 (45)	107 (59)	0.002
Diabetes mellitus n (%)	117 (27)	77 (42)	< 0.001
Hyperlipidemia n (%)	94 (14)	31 (17)	0.351
Current smoking n (%)	210 (32)	41 (22)	0.015
Previous CAD n (%)	183 (28)	66 (26)	0.026
Prior stroke/TIA n (%)	21 (3)	19 (10)	< 0.001
Type of ACS n (%)			
STEMI	419 (63)	105 (57)	0.151
NSTEMI	179 (27)	62 (34)	0.102
UA	56 (8)	13 (7)	0.557
Major bleeding n (%)	14 (2)	9 (5)	0.039
Killip class ≥2 n (%)	33 (5)	48 (26)	< 0.001
Medication at discharge			
Beta-blocker n (%)	580 (88)	136 (74)	< 0.001
Statin n (%)	539 (81)	143 (78)	0.339
ACE-I/ARB n (%)	555 (84)	126 (99)	< 0.001
Outcomes			
In-hospital death n (%)	0 (0)	30 (16)	< 0.001
Stroke n (%)	14 (2)	9 (5)	0.039
HF admission n (%)	24 (4)	25 (14)	< 0.001
Myocardial reinfarction n (%)	62 (9)	17 (9)	0.980
TVR n (%)	78 (12)	11 (6)	0.025
Cardiac death n (%)	0 (0)	59 (32)	< 0.001

*HF* heart failure, *CAD* coronary artery disease, *TIA* transient ischemic attack, *ACE-I* angiotensin-converting enzyme inhibitors, *ARB* angiotensin receptor blocker, *ACS* acute coronary syndrome, *UA* unstable angina, *NSTEMI* non-ST-elevation myocardial infarction, *STEMI* ST-elevation myocardial infarction, *TVR* target vessel revascularization

Non-survivors were older (67 ± 12 vs 62 ± 12 years, *p* <  0.001) and had a higher prevalence of diabetes mellitus (DM) (42 vs 27%, *p* <  0.001). Compared with survivors, history of heart failure, hypertension (HT), previous coronary artery disease (CAD), and higher Killip class were more frequent in non-survivors. On the other hand, use of beta-blockers and angiotensin-converting enzyme inhibitors was lower in non-survivors than survivors (Table 1). Major bleeding rates were higher in non-survivors than survivors (5% vs 2%, *p* = 0.039).

### Laboratory findings

The laboratory variables of the groups are shown in Table 2. LVEF was significantly lower in non-survivors than survivors (41.3 ± 11.8% vs 47.5 ± 10.0%, *p* <  0.001). Non-survivors had higher leukocyte counts and higher levels of sCr than survivors. Moreover, INR and TB level were higher in non-survivors compared with survivors. Serum troponine level was comparable between groups (Table 2).

**Table 2:** Laboratory results of the study groups

Variable	Survivors (*n* = 663)	Non-survivors (*n* = 183)	*P* value
Peak-troponin-Ia, ng/mL	28 (19–44)	30 (18–51)	0.444^a^
Peak-troponin-I^*^, ng/mL	1.8 (0.6–4.2)	2.3 (0.5–12.4)	0.853^b^
Total cholesterol	170 ± 40	179 ± 46	0.128
SCr^*^_adm_ (mg/dl)	0.82 (0.73–1.02)	1.03 (0.79–1.42)	< 0.001
WBC (× 10^3^/mm^3^)	11 ± 3	12 ± 4	< 0.001
Hemoglobin (g/dl)	12.6 ± 2	11.8 ± 2.2	< 0.001
LVEF (%)	47.5 ± 10.0	41.3 ± 11.8	< 0.001
ALT^*^ (U/L)	32 (21–49)	28 (18–54)	0.420
AST^*^ (U/L)	51 (27–105)	44 (23–129)	0.321
Total bilirubin^*^ (mg/dl)	0.57 (0.40 ± 0.78)	0.60 (0.40–0.90)	0.015
INR	1 ± 0.11	1 ± 0.16	< 0.001
MELD score	7.8 ± 2.4	10.1 ± 4.4	< 0.001

*SCr* serum creatinine at admission, *WBC* wight blood cell, *LVEF* left ventricular ejection fraction, *ALT* alanine transaminase, *AST* aspartat transaminase, *INR* international normalised ratio, *MELD* model for liver end-stage liver disease

^*^Comparison was made using Mann-Whitney *U* test at *P* < 0.05, and these values were described by median with inter-quartile range (25th and 75th percentile)

^a^Comparison was made in patients with ST-elevation myocardial infarction

^b^Comparison was made in patients with non-ST-elevation myocardial infarction

Compared with survivors, MELD score was higher in non-survivors (10.1 ± 4.4 vs. 7.8 ± 2.4, *p* <  0.001). In the correlation analysis, MELD score was inversely and weakly correlated with LVEF (*r* = − 0.19, *p* <  0.001), and hemoglobin (*r* = − 0.25, *p* <  0.001), but positively correlated with age (*r* = 0.28, *p* <  0.001).

### Angiographic and procedural characteristics

The angiographic and procedural characteristics of the patients are provided in Table 3. Stent use, stent type, and tirofiban use did not differ significantly between the two groups, whereas the rate of multivessel disease was more frequent in non-survivors than survivors (60 vs 45%, *p* <  0.001).

**Table 3:** Angiographic and procedural characteristics of the study population

Variable	Survivors (*n* = 663)	Non-sruvivors (*n* = 183)	*P*-value
Vessel involvement			0.374
LMCA	0(0)	1 (0.6)	
LAD	304 (46)	76 (42)	
CX	99 (15)	23 (13)	
RCA	209 (32)	65 (36)	
Others	51 (8)	19 (10)	
Multi-vessel disease n (%)	296 (45)	110 (60)	< 0.001
Stent use n (%)	634 (96)	173 (95)	0.533
Stent length. mm	21 (18–28)	23 (18–28)	0.722
Stent diameter, mm	3.4 ± 0.6	3.5 ± 0.6	0.887
Stent type			0.141
DES n (%)	67 (10)	10 (6)	
BMS n (%)	583 (90)	168 (94)	
Tirofiban use n (%)	257 (39)	66 (36)	0.506

*LMCA* left main coronary artery, *LAD* left anterior descending coronary artery, *CX* circumflex coronary artery, *RCA* right coronary artery, *DES* drug-eluting stent, *BMS* bare-metal stent

### MELD score, LVEF, and clinical outcomes

Table 1 presents the clinical outcomes. Sixteen percent of total deaths was in-hospital death and 32% was due to cardiac causes. Stroke/TIA rate was more prevalent in non-survivors than survivors (5% vs. 2%, *p* = 0.039). Hospitalization for heart failure was also higher in non-survivors than survivors (14% vs. 4%, *p* <  0.001), however TVR rate was lower in non-survivors (12% vs. 6%, *p* = 0.025). Myocardial reinfarction rate was comparable in the groups.

The independent predictors for all-cause death identified using the multivariate Cox regression analysis are presented in Table 4. MELD score and LVEF were independently predictive for all-cause mortality (HR: 1.116, 95%CI: 1.069–1.164, *p* <  0.001; HR: 0.972, 95%CI: 0.958–0.986, *p* <  0.001, respectively, Table 4).

**Table 4:** Independent predictors of all-cause mortality

Variable	Univariate			Multivariate		
	HR	95% CI	*p*-value	HR	95% CI	*p*-value
Age (per 1 year)	1.042	1.029–1.056	< 0.001	1.023	1.008–1.038	0.002
Male	0.663	0.486–0.904	0.009	0.944	0.666–1.354	0.774
Diabetes mellitus	1.780	1.327–2.387	< 0.001	1.384	1.004–1.907	0.047
Hipertension	1.480	1.103–1.986	0.009	0.999	0.719–1.390	0.997
Stroke history	2.602	1.617–4.189	< 0.001	1.954	1.193–3.200	0.008
History of CAD	1.370	1.013–1.852	0.011	1.113	0.804–1.542	0.518
Major bleeding	1.898	0.970–3.713	0.068	0.812	0.395–1.669	0.571
Multi-vessel disease	1.872	1.392–2.518	< 0.001	1.197	0.866–1.654	0.276
Killip class ≥2	5.545	3.981–7.722	< 0.001	4.149	2.907–5.922	< 0.001
LVEF (per 1% change)	0.957	0.945–0.969	< 0.001	0.972	0.958–0.986	< 0.001
Hemoglobin (per 1 mg/dl)	0.805	0.748–0.866	< 0.001	0.887	0.816–0.965	0.005
WBC (per 10^3^/L)	1.081	1.043–1.121	< 0.001	1.063	1.024–1.103	0.001
B-blocker use at follow-up	0.489	0.351–0.682	< 0.001	0.638	0.444–0.917	0.015
ACE/ARB use at follow-up	0.452	0.331–0.619	< 0.001	0.989	0.668–1.464	0.956
TVR	0.490	0.266–0.902	0.022	0.765	0.410–1.4128	0.401
MELD^a^ score (per 1 point)	1.291	1.222–1.364	< 0.001	1.116	1.069–1.164	< 0.001

*HR* hazard ratio, *CI* confidence interval, *LVEF* left ventricular ejection fraction, *MELD* model for end-stage liver disease, *WBC* white blood cell, *HDL-C* high-density lipoprotein cholesterol, *ACE-I/ARB* angiotensin-converting enzyme inhibitors/ angiotensin-reseptor blocker, *TVR* target vessel revascularization

^a^Considered as continous variable

**Fig. 1:**
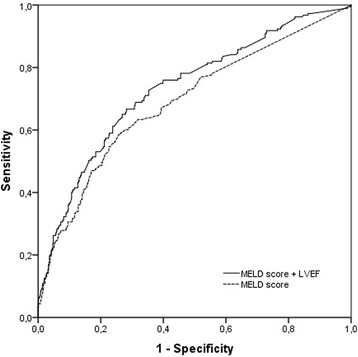
Receiver operating characteristic (ROC) curves for the MELD score alone and the combining MELD score with LVEF for predicting all-cause total mortality

**Fig. 2:**
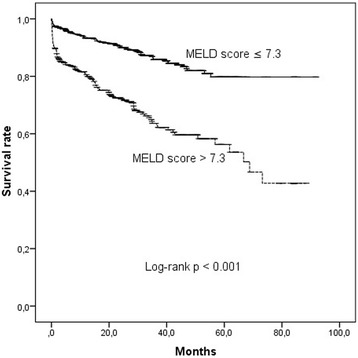
Kaplan-Meier survival curves of all-cause mortality according to the MELD score

AUC of LEVF for all-cause mortality was 0.659 (0.612–0.715, *p* < 0.001). The analysis of ROC curve showed an area under curve (AUC) of 0.690 for the prediction of all-cause mortality by MELD score of 7.3 (Fig. 1). The patients were divided into two subgroups based on this cut-point of MELD score; low (≤ 7.3) and high-subgroups (> 7.3). In subgroup analyses, in-hospital death (3 vs 0.6%, *p* < 0.001), cardiac death (5 vs 1.5%, *p* < 0.001), and all-cause total mortality (14 vs 18%, *p* < 0.001, Fig. 2) were higher in patients with high MELD score than those with low MELD score. Morever, heart failure admission rate was higher in high-subgroups than low-subgroups (4 vs 2%, *p* < 0.001). There was no significant difference between groups with regard to myocardial reinfarction, stroke/TIA, and TVR rates (5 vs 5%, 7 vs 5%, 2 vs 1%, and 7 vs 4%, respectively, each *p* > 0.05). Compared with the MELD score alone, the combining LVEF with MELD score was associated significant improvement in the ability to predict mortality (AUC:0.733 vs 0.690, *p* < 0.001, Fig. 1). The addition of LVEF to MELD score significantly improved the reclassification (NRI = 24.6%, Table 5) and the integrated discrimination (IDI: 0.045, *p* < 0.001).

**Table 5:** Reclassification of ACS patients who died or who were alive at follow-up based on LVEF status

MELD score without LVEF		MELD score with LVEF		Total
	< 10% risk	10–30% risk	> 30% risk	
Patients who died, no.				
< 10% risk	0	0	0	0
10–30% risk	14	86	31	131
> 30% risk	0	7	45	52
Total no.	14	93	76	183
Patients who were alive, no.				
< 10% risk	0	0	0	0
10–30% risk	149	416	38	603
> 30% risk	0	16	44	60
Total	149	432	82	663

*ACS* acute coronary syndrome, *MELD* the Model for End-Stage Liver Disease, *LVEF* left ventricular ejection fraction

## Discussion

This study demonstrated that MELD score and LVEF were associated with increased all-cause mortality in ACS patients treated with PCI who were not on anticoagulant therapy during the median follow-up of 28 months. To the best of our knowlodge, this is the first study investigating the combining of LVEF with MELD score for predicting mortality in these patients. Morever, the present study showed that the combined use of LVEF and MELD score was better able to predict all-cause mortality compared with the MELD score alone.

Bilirubin, the end product of heme catabolism, is derived primarily from circulating hemoglobin [19]. Although bilirubin has long been considered a waste product, it is currently recognized as a potent endogenous antioxidant which has the capacity to reduce the reactive oxygen radicals and, prevent the oxidation of low-density lipoprotein cholesterol [20]. A growing number of studies report a negative association between serum bilirubin levels and the prevalence of CAD [21]. Higher serum bilirubin levels were associated with lower Framingham risk scores [21].

The above-mentioned studies were not performed under acute stress condition. On the other hand, heme oxygenase (HO) 1 enzyme activity and its end product bilirubin increase with acute stress [22]. Also, HO-1 levels have a positive correlation with TB levels in patients with acute MI [22]. Celik et al. investigated associations of TB level with the devolopment of post-PCI coronary no-reflow and in-hospital major adverse cardiac events (MACE) [8]. They demonstrated that serum bilirubin levels were independently associated with no-reflow and in-hospital MACE in STEMI patients undergoing PCI. However, in their study, there was no association between TB levels and long-term mortality. In another study by Kaya et al., TB levels were found to be related to severity of coronary artery disease in patients with NSTEMI [23]. They showed that its level was independently associated with high SYNTAX score. In our study, non-survivors had a higher levels of TB compared with survivors. Also, TB was an independent predictor of all-cause mortality at follow-up.

sCr levels has a significant prognostic value in ACS patients. It has been shown that baseline renal dysfunction was associated with a higher mortality in patients with ACS as found in our study [24]. Similarly, renal dysfunction has been shown to be independently associated with mortality STEMI patients treated with primary PCI [25]. Several factors associated with impaired renal function may contribute to the adverse outcome of patients with acute coronary syndrome. These factors include insulin resistance [26], alterations in the extracellular matrix [27], oxidative stress [28], inflammation [29], endothelial dysfunction [30], reninangiotensin- aldosterone system activation [31], and increased plasma levels of fibrinogen and homocysteine [32]. Also, derangements in calcium–phosphate homeostasis and anemia may increase cardiovascular risk by renal dysfunction [33]. All of them are asssociated with accelerated atherosclerosis and endothelial dysfunction. Furthermore, patients with renal dysfunction have a higher prevalence of baseline cardiovascular comorbidities such as diabetes, heart failure, previous MI and stroke and coronary interventions [34]. In addition, diffuse coronary artery disease proven by angiography was more frequent in these patients. All these conditions may related to adverse prognosis in patienst with ACS [35].

A higher INR in the absence of anticogulant use was associated with 6-month mortality in acute PE patients [10, 11]. INR > 1.2 was independent predictor of mortality in those patients. Okada et al. showed an increased INR was independent predictor of all-cause mortality in acute heart failure patients without anticoagulant therapy [10]. In their study, INR > 1.05 was significantly related to mortality. Similarly, an elevated INR was independent predictor of mortality in our population not on anticoagulant therapy. Increased INR may be associated with activated coagulation, inflammation, neurohumoral activation, and hepatic insufficiency [10]. Also, it may represent a serious inflammatory state in ACS.

Prior studies have described an relation EF and advers outcomes after ACS [36]. In a recent study by Wei et al., they demonstrated that LVEF was an independent predictor of in-hospital and 1-year mortality in STEMI patients [37]. It has been shown that LVEF independently predicted major adverse cardiac events in STEMI patients [38]. Similarly, a low LVEF was found to have predictive power for in patients with NSTEMI [39].

As MELD score requires 3 parameters only, it is the simplest score. Morever, serum TB, Cr, and INR can readily obtanied by an easily-accessible and non-invasive blood test and objectively evaluated. Similarly, LVEF can be easily measured with a bedside echocardiogram. Furthermore, these laboratory parameters indicating cardiac, hepatic and renal dysfunction can be associated with mortality in cardiovascular disease as in the aforementioned studies. In our study, non-survivors had a higher MELD score than survivors. Also, stroke/TIA and heart failure admission rates were higher non-survivors compared with survivors, whereas there was no significant difference in rate of myocardial reinfarction between non-survivors and survivors. The patients with a higher MELD score had a higher rate of cardiac death compared with those with low MELD score in our study.

Our study has several limitations. The database analysis is retrospective in nature and therefore has all the associated limitations of a retrospective study. The study can not establish causal relationships and is subject to inherent biases. Also, we did not measure the level of specific coagulation factors such as factor II,VII, and IX in these patients. Contrary to the previous studies, this cut-point used to predict mortality in present study was not consistent with what has been used in the surgical literature [40, 41]. As the current study included patients with ACS, which is a different clinical setting from the reported clinical situation in the previous literature, this may explain the difference in the cut-point used in our study. Thus, further studies are required to validate the prognostic performance and optimal cutoff values of the MELD score in patients with ACS. It has been shown that troponine- I as myocardial injury marker, and Brain Natriuretic Peptide (BNP) as stress biomarker were associated with mortality in both patients with normal LVEF and heart failure [42, 43]. In present study, although troponine-I level was measured, we did not measure the serum level of BNP. Therefore, we did not assess relation of this marker to clinical outcomes. In our study, patients with right ventricle dysfunction or right ventricular dilatation were excluded from this study. Therefore, association hepatic dysfunction with right ventricle was not evaluated. Also, we did not evaluated the association between depressed EF and hepatic dysfunction in this study. Another limitation is that syntax score indicating complexity of coronary artery lesions was not used in the present study. Last, DM was associated with mortality in our study. The DM patients treated with incretin had a significantly lower rate of major cardiovascular events compared to those were not treated by this treatment [44, 45]. As data regarding incretin usage was not present in many patients, its effect on mortality in present study could not be assesed.

## Conclusions

The MELD score is a simple score derived from an easily-accessible and non-invasive blood test. Similarly, LVEF may be easily determined by a bedside echocardiogram. They were independently associated with all-cause mortality in ACS patients undergoing PCI who were not receiving previous anticoagulant therapy. Furthermore, adding LVEF to MELD score improved the predictive value for all-cause mortality in these patients.

## Additional files

Additional file 1: Subgroup analysis according to gender (Tables S6-S8). (ZIP 27 kb)

Additional file 2: Subgroup analysis according to age (Tables S9-S11). (ZIP 27 kb)

## Abbreviations

ACS: Acute coronary syndrome
BNP: Brain natriuretic peptide
CAD: Coronary artery diseases
CCS: Canadian Cardiovascular Society
DM: Diabetes mellitus
HO 1: Heme oxygenase 1
HT: Hypertension
IDI: Integrated discrimination improvement
INR: International normalized ratio
LBBB: Left bundle branch block;
LVEF: Left ventricular ejection fraction
MACE: Major adverse cardiac events
MELD: Model for End-Stage Liver Disease
NRI: Net reclassification improvement
NSTEMI: Non-ST elevation myokard infarction
PCI: Percutaneous coronary intervention
PE: Pulmonay embolism
RA: Right atrial
ROC: receivers operating characteristic
RV: Right ventricle
sCr: Serum creatinine
STEMI: ST-elevation myocardial infarction
TB: Total bilirubin
TİA: transient ischemic attack
TR: Tricuspid regurjitation
TVR: Targetvessel revascularization
UA: Unstable angina
