# Supplementary material for: Combined value of left ventricular ejection fraction and the Model for End-Stage Liver Disease (MELD) score for predicting mortality in patients with acute coronary syndrome who were undergoing percutaneous coronary intervention
Source: BMC Cardiovasc Disord. 2018 Mar 2;18:44. doi: 10.1186/s12872-018-0782-8 (PMC5833061; doi:10.1186/s12872-018-0782-8)
Supplement: Supplementary file 1 — Subgroup analysis according to gender (Tables S6-S8). (ZIP 27 kb) [file 12872_2018_782_MOESM1_ESM.zip › table 6.supplR3.doc]

**Table S6. Baseline characteristics of the study population.**

|  | **Variable** | **Male (n = 629)** | **Female(n= 217)** | | **P-value** | |  | |
| --- | --- | --- | --- | --- | --- | --- | --- | --- |
|  | Age (year) | 60 ± 12 | 67 ± 12 | | < 0.001 | |  | |
|  | Female n (%)  History of HF n (%) | 0 (0)  15 (2) | 217 (100)  11 (5) | | N.A.  0.048 | |  | |
|  | Hypertension n (%) | 262 (42) | 146 (67) | | < 0.001 | |  | |
|  | Diabetes mellitus n (%) | 156 (25) | 98 (45) | | < 0.001 | |  | |
|  | Hyperlipidemia n (%) | 84 (13) | 41 (19) | | 0.047 | |  | |
|  | Current smoking n (%) | 220 (35) | 31 (14) | | < 0.001 | |  | |
|  | Previous CAD n (%) | 187 (30) | 62 (29) | | 0.747 | |  | |
|  | Prior stroke/TIA n (%) | 28 (5) | 12 (6) | | 0.519 | |  | |
|  | Type of ACS n (%)  STEMI  NSTEMI  UA | 398 (63)  173 (28)  48 (8) | 126 (58)  68 (31)  21 (10) | | 0.173  0.281  0.342 | |  | |
|  | Major bleeding n (%) | 13 (2) | 10 (5) | | 0.047 | |  | |
|  | Killip class ≥ 2 n (%) | 53 (8) | 28 (53) | | 0.053 | |  | |
|  | Medication at discharge |  |  | |  | |  | |
|  | Beta-blocker n (%) | 541 (86) | 175 (81) | | 0.059 | |  | |
|  | Statin n (%)  ACE-I/ARB n (%) | 539 (81)  512 (81) | 143 (78)  169 (78) | | 0.339  0.259 | |  | |
| Outcomes  Total mortalite n (%) | | 124 (20) | 59 (27) | | 0.021 | |  | |
| In-hospital death n (%) | | 15 (2) | 15 (7) | 0.002 | |  | |  |
| Stroke n (%) | | 17 (3) | 6 (3) | 0.961 | |  | |  |
| HF admission n (%) | | 32 (5) | 17 (8) | 0.135 | |  | |  |
| Reinfarction n (%) | | 54 (9) | 25 (12) | 0.200 | |  | |  |
| TVR n (%) | | 63 (10) | 26 (12) | 0.416 | |  | |  |
| Cardiac death n (%) | | 37 (6) | 22 (10) | 0.034 | |  | |  |

HF: heart failure, CAD; coronary artery disease, TIA: transient ischemic attack, ACE-I: angiotensin-converting enzyme inhibitors, ARB: angiotensin receptor blocker, ACS: acute coronary syndrome, UA: unstable angina, NSTEMI: non-ST-elevation myocardial infarction, STEMI: ST-elevation myocardial infarction, TVR; target vessel revascularization.
