# Supplementary material for: Combined value of left ventricular ejection fraction and the Model for End-Stage Liver Disease (MELD) score for predicting mortality in patients with acute coronary syndrome who were undergoing percutaneous coronary intervention
Source: BMC Cardiovasc Disord. 2018 Mar 2;18:44. doi: 10.1186/s12872-018-0782-8 (PMC5833061; doi:10.1186/s12872-018-0782-8)
Supplement: Supplementary file 1 — Subgroup analysis according to gender (Tables S6-S8). (ZIP 27 kb) [file 12872_2018_782_MOESM1_ESM.zip › Table 7.supplR3.doc]

**Table S7. Laboratory results of the study groups.**

| **Variable** | **Male (n = 629)** | **Female (n = 217)** | **P value** |
| --- | --- | --- | --- |
| Peak-troponin-I*, ng/mL | 28 (21-50) | 26 (17-39) | 0.451^¶^ |
| Peak-troponin-I*, ng/mL | 2.0 (0.53-8.0) | 2.0 (0.6-2.8) | 0.963^£^ |
| Total cholesterol  SCr* _adm_ (mg/dl) | 177 ± 44  0.85 (0.75-1.08) | 176 ± 46  0.87 (0.70-1.10) | 0.707  0.054 |
| WBC (x10³/mm³) | 11 ± 4 | 11 ± 4 | 0.977 |
| Hemoglobin (g/dl) | 13.0 ± 2 | 11.0 ± 1.7 | < 0.001 |
| LVEF (%) | 46.0 ± 10.7 | 46.4 ± 10.8 | 0.676 |
| ALT* (U/L) | 34 (21-52) | 24 (17-41) | <0.001 |
| AST* (U/L) | 52 (29-111) | 43 (21-96) | 0.097 |
| Total bilirubin* (mg/dl) | 0.60 (0.40 -0.80 ) | 0.50 (0.40-0.70) | 0.001 |
| INR | 1 ± 0.12 | 1 ± 0.13 | 0.767 |
| MELD score | 8.2 ± 3.1 | 8.3 ± 3.0 | 0.766 |

SCr; serum creatinine at admission, WBC; wight blood cell, LVEF: left ventricular ejection fraction, ALT; alanine transaminase, AST; aspartat transaminase, INR; international normalised ratio, MELD; model for liver end-stage liver disease.

* Comparison was made using Mann-Whitney *U* test at *P* <0.05, and these values were described by median with inter-quartile range ( 25^th^ and 75^th^ percentile).

^¶^ Comparison was made in patients with ST-elevation myocardial infarction.

^£^ Comparison was made in patients with non-ST-elevation myocardial infarction.
