# Supplementary material for: Combined value of left ventricular ejection fraction and the Model for End-Stage Liver Disease (MELD) score for predicting mortality in patients with acute coronary syndrome who were undergoing percutaneous coronary intervention
Source: BMC Cardiovasc Disord. 2018 Mar 2;18:44. doi: 10.1186/s12872-018-0782-8 (PMC5833061; doi:10.1186/s12872-018-0782-8)
Supplement: Supplementary file 1 — Subgroup analysis according to gender (Tables S6-S8). (ZIP 27 kb) [file 12872_2018_782_MOESM1_ESM.zip › table 8.supplR3.doc]

**Table S8. Angiographic and procedural characteristics of the study population.**

| **Variable** | **Male (n = 629)** | **Female (n = 217)** | **P-value** |
| --- | --- | --- | --- |
| Vessel involvement |  |  | 0.028 |
| LMCA  LAD  CX  RCA  Others | 0(0)  275 (44)  94 (15)  199 (32)  62 (10) | 1 (0.6)  175 (49)  28 (13)  75 (35)  7 (4) |  |
| Multi-vessel disease n (%) | 292 (46) | 114 (53) | 0.103 |
| Stent use n (%) | 603 (96) | 207 (95) | 0.578 |
| Stent length*, mm | 20 (18-28) | 22 (18-28) | 0.590 |
| Stent diameter, mm | 3.5 ± 0.6 | 3.3 ± 0.5 | < 0.001 |
| Stent type |  |  | 0.543 |
| DES n (%) | 61 (10) | 16 (8) |  |
| BMS n (%) | 559 (90) | 193 (92) |  |
| Tirofiban use n (%) | 260 (41) | 63 (29) | 0.002 |
|  |  |  |  |

LMCA; left main coronary artery, LAD; left anterior descending coronary artery, CX; circumflex coronary artery, RCA; right coronary artery, DES; drug-eluting stent, BMS; bare-metal stent.

*Comparison was made using Mann-Whitney *U* test at *P* <0.05, and these values were described by median with inter-quartile range ( 25^th^ and 75^th^ percentile).
