# Supplementary material for: Combined value of left ventricular ejection fraction and the Model for End-Stage Liver Disease (MELD) score for predicting mortality in patients with acute coronary syndrome who were undergoing percutaneous coronary intervention
Source: BMC Cardiovasc Disord. 2018 Mar 2;18:44. doi: 10.1186/s12872-018-0782-8 (PMC5833061; doi:10.1186/s12872-018-0782-8)
Supplement: Supplementary file 2 — Subgroup analysis according to age (Tables S9-S11). (ZIP 27 kb) [file 12872_2018_782_MOESM2_ESM.zip › Table 10.supplR3.doc]

**Table S10. Laboratory results of the study groups.**

| **Variable** | **Age < 65 years**  **(n = 487)** | **Age ≥ 65 years**  **(n = 359)** | **P value** |
| --- | --- | --- | --- |
| Peak-troponin-I*, ng/mL | 28 (20-47) | 28 (18-44) | 0.829^¶^ |
| Peak-troponin-I*, ng/mL | 2.0 (0.6-4.3) | 1.7 (0.6-5.1) | 0.651^£^ |
| Total cholesterol | 185 ± 48 | 166 ± 38 | < 0.001 |
| SCr* _adm_ (mg/dl) | 0.80 (0.72-0.94) | 0.96 (0.78-1.30) | < 0.001 |
| WBC (x10³/mm³) | 11 ± 4 | 11 ± 4 | < 0.001 |
| Hemoglobin (g/dl) | 13.0 ± 2 | 11.7 ± 1.8 | < 0.001 |
| LVEF (%) | 46.5 ± 10.7 | 45.6 ± 10.8 | 0.217 |
| ALT* (U/L) | 34 (22-53) | 27 (19-46) | 0.002 |
| AST* (U/L) | 51 (27-113) | 48 (27-98) | 0.336 |
| Total bilirubin* (mg/dl) | 0.56 (0.40 ± 0.80 ) | 0.60 (0.40-0.80) | 0.067 |
| INR | 1 ± 0.12 | 1 ± 0.13 | < 0.001 |
| MELD score | 7.8 ± 2.9 | 8.9 ± 3.3 | < 0.001 |

SCr; serum creatinine at admission, WBC; wight blood cell, LVEF; left ventricular ejection fraction, ALT; alanine transaminase, AST; aspartat transaminase, INR; international normalised ratio, MELD; model for liver end-stage liver disease.

* Comparison was made using Mann-Whitney *U* test at *P* <0.05, and these values were described by median with inter-quartile range ( 25^th^ and 75^th^ percentile).

^¶^ Comparison was made in patients with ST-elevation myocardial infarction.

^£^ Comparison was made in patients with non-ST-elevation myocardial infarction.
