# Supplementary material for: Combined value of left ventricular ejection fraction and the Model for End-Stage Liver Disease (MELD) score for predicting mortality in patients with acute coronary syndrome who were undergoing percutaneous coronary intervention
Source: BMC Cardiovasc Disord. 2018 Mar 2;18:44. doi: 10.1186/s12872-018-0782-8 (PMC5833061; doi:10.1186/s12872-018-0782-8)
Supplement: Supplementary file 2 — Subgroup analysis according to age (Tables S9-S11). (ZIP 27 kb) [file 12872_2018_782_MOESM2_ESM.zip › Table 11.supplR3.doc]

**Table S11. Angiographic and procedural** characteristics of the study population.

| **Variable** | **Age < 65 years**  **(n = 487)** | **Age ≥ 65 years**  **(n = 359)** | **P-value** |
| --- | --- | --- | --- |
| Vessel involvement |  |  | 0.002 |
| LMCA  LAD  CX  RCA  Others | 0(0)  239 (49)  75 (15)  133 (27)  40 (8) | 1 (0.3)  141 (39)  47 (13)  41 (39)  29 (8) |  |
| Multi-vessel disease n (%) | 202 (42) | 204 (57) | < 0.001 |
| Stent use n (%) | 468 (96) | 339 (94) | 0.252 |
| Stent length *, mm | 21 (18-28) | 22 (18-29) | 0.920 |
| Stent diameter, mm | 3.4 ± 0.6 | 3.5 ± 0.6 | 0.029 |
| Stent type |  |  | 0.378 |
| DES n (%) | 49 (10) | 28 (8) |  |
| BMS n (%) | 429 (90) | 322 (92) |  |
| Tirofiban use n (%) | 201 (41) | 122 (34) | 0.031 |
|  |  |  |  |

LMCA; left main coronary artery, LAD; left anterior descending coronary artery, CX; circumflex coronary artery, RCA; right coronary artery, DES; drug-eluting stent, BMS; bare-metal stent.

* Comparison was made using Mann-Whitney *U* test at *P* <0.05, and these values were described by median with inter-quartile range ( 25^th^ and 75^th^ percentile).
