# Supplementary material for: Combined value of left ventricular ejection fraction and the Model for End-Stage Liver Disease (MELD) score for predicting mortality in patients with acute coronary syndrome who were undergoing percutaneous coronary intervention
Source: BMC Cardiovasc Disord. 2018 Mar 2;18:44. doi: 10.1186/s12872-018-0782-8 (PMC5833061; doi:10.1186/s12872-018-0782-8)
Supplement: Supplementary file 2 — Subgroup analysis according to age (Tables S9-S11). (ZIP 27 kb) [file 12872_2018_782_MOESM2_ESM.zip › Table 9.supplR3.doc]

**Table S9. Baseline characteristics of the study population.**

|  | **Variable** | **Age < 65 years**  **(n = 487)** | | **Age ≥ 65 years**  **(n = 359 )** | | **P-value** | | |  |
| --- | --- | --- | --- | --- | --- | --- | --- | --- | --- |
|  | Age (year) | 53 ± 8 | | 74 ± 6 | | < 0.001 | | |  |
|  | Female n (%)  History of HF n (%) | 82 (17)  13 (3) | | 134 (37)  13 (4) | | < 0.001  0.428 | | |  |
|  | Hypertension n (%) | 195 (40) | | 213 (59) | | < 0.001 | | |  |
|  | Diabetes mellitus n (%) | 132 (27) | | 122 (34) | | 0.031 | | |  |
|  | Hyperlipidemia n (%) | 80 (16) | | 45 (13) | | 0.115 | | |  |
|  | Current smoking n (%) | 189 (39) | | 62 (17) | | < 0.001 | | |  |
|  | Previous CAD n (%) | 136 (28) | | 113 (32) | | 0.263 | | |  |
|  | Prior stroke/TIA n (%) | 15 (3) | | 25 (7) | | < 0.001 | | |  |
|  | Type of ACS n (%)  STEMI  NSTEMI  UA | 315 (65)  124 (26)  40 (8) | | 209 (58)  117 (33)  29 (8) | | 0.056  0.023  0.943 | | |  |
|  | Major bleeding n (%) | 16 (3) | | 7 (2) | | 0.238 | | |  |
|  | Killip class ≥ 2 n (%) | 41 (8) | | 40 (11) | | 0.183 | | |  |
|  | Medication at discharge |  | |  | |  | | |  |
|  | Beta-blocker n (%) | 439 (90) | | 277 (77) | | < 0.001 | | |  |
|  | Statin n (%)  ACE-I/ARB n (%) | 404 (83)  417 (86) | | 278 (77)  264 (74) | | 0.045  <0.001 | | |  |
| Outcomes | | |  | |  | |  | | |
| In-hospital death n (%) | | | 12 (3) | | 18 (5) | | 0.047 |  | |
| Stroke n (%) | | | 13 (3) | | 10 (3) | | 0.918 |  | |
| HF admission n (%) | | | 19 (4) | | 30 (8) | | 0.006 |  | |
| Myocardial reinfarction n (%) | | | 47 (10) | | 32 (9) | | 0.716 |  | |
| TVR n (%) | | | 57 (12) | | 32 (9) | | 0.191 |  | |
| Cardiac death n (%) | | | 24 (5) | | 35 (10) | | 0.007 |  | |
| All-cause mortalite n (%) | | | 75 (15) | | 108 (30) | | <0.001 |  | |
|  |  | | | | | | | | |

HF: heart failure, CAD; coronary artery disease, TIA: transient ischemic attack, ACE-I: angiotensin-converting enzyme inhibitors, ARB: angiotensin receptor blocker, ACS: acute coronary syndrome, UA: unstable angina, NSTEMI: non-ST-elevation myocardial infarction, STEMI: ST-elevation myocardial infarction, TVR; target vessel revascularization.
